# A NEET distinction: youths not in employment, education or training follow different pathways to illness and care in psychosis

**DOI:** 10.1007/s00127-018-1565-3

**Published:** 2018-08-09

**Authors:** Srividya Iyer, Sally Mustafa, Geneviève Gariépy, Jai Shah, Ridha Joober, Martin Lepage, Ashok Malla

**Affiliations:** 1 Department of Psychiatry, McGill University, Montreal, Canada; 2 Prevention and Early Intervention Program for Psychosis (PEPP), Douglas Mental Health University Institute, Montreal, Canada; 3 ACCESS Open Minds (pan-Canadian youth mental health services research network), Douglas Mental Health University Institute, Montreal, Canada

**Keywords:** Psychosis, Early intervention, NEET, Treatment delays, Pathways to care, Prodrome, Negative symptoms

## Abstract

**Purpose:**

The early phases of psychosis, including the prodrome, often feature educational/occupational difficulties and various symptoms and signs, that can render or keep youths “Not in Employment, Education or Training” (NEET). Conversely, NEET status itself may increase risk for illness progression and impaired functioning, and impede access to appropriate services for psychosis. As these issues have not been investigated, we aimed to examine differences in the illness and care pathways and characteristics of youths with psychosis who are NEET and non-NEET.

**Methods:**

Youths entering a catchment-based Canadian early intervention service for psychosis (N = 416) were assessed as being NEET or non-NEET and compared on symptomatology, premorbid adjustment, prodrome and duration of untreated psychosis (DUP).

**Results:**

Thirty-nine percent of the sample was NEET. Compared to non-NEET youths, NEET youths had 34% higher negative symptoms scores, longer prodromes (median of 52 weeks vs. 24 weeks), and were more often continuously ill after their first psychiatric change until the onset of psychosis (62% vs. 45%). Both groups had similar premorbid adjustment scores until late adolescence when scores were significantly worse for NEET youths. Accounting for other predictors, NEET youths had 23% longer DUPs on average, despite having made more help-seeking attempts.

**Conclusions:**

Despite being more narrowly defined, NEET status was thrice as prevalent in our sample as in the Canadian population. The NEET group followed a distinct trajectory of persistent symptoms and functional decline before presenting with a psychotic disorder. The systemic delays that NEET youths encountered indicate a need for better-targeted early identification efforts.

## Introduction

The term NEET, coined in the United Kingdom [1], is now used widely to refer to young people who are “Not in Education, Employment or Training”. There has been growing concern about this group, given its high risk for adverse economic, health, legal and psychosocial outcomes [2–6]. In 2016, the proportion of individuals aged 15–29 who were NEET ranged from 5.3–28% (average 13.9%) in Organisation for Economic Co-operation and Development (OECD) countries [5]. The phase of life critical for educational and employment milestones is also one in which individuals are particularly vulnerable to the onset of mental disorders [7, 8]. The relationship between NEET status and mental illness may therefore be bi-directional [9, 10]: while being NEET elevates the risk for mental disorders, becoming NEET may also be a consequence of emerging or pre-existing mental illnesses. In their study based on a national cohort of 18-year olds in the United Kingdom, Goldman-Mellor et al. [10] found that NEET youths were substantially more likely to have experienced mental health problems in childhood/adolescence than their non-NEET counterparts (60% vs. 35%, respectively). Notably, NEET youths also had higher rates of concurrent mental health problems, independent of their pre-existing mental ill-health vulnerability. Cross et al. [11] found that NEET status was a significant transdiagnostic predictor of progression from subthreshold to threshold presentations of major mental disorders. However, their 1-year follow-up study could not disentangle whether NEET status was a consequence of mental and substance use problems, or a risk factor for illness progression (with non-NEET status being conversely a protective factor), or both.

In psychosis, functional decline often precedes overt clinical signs during the prodrome [12, 13]. While there have been calls [9] for research into the intersection between functional decline, mental disorders and NEET status, this overlap has not been systematically investigated, especially in psychosis. It is important to examine NEET status in the context of psychosis, an illness associated with adverse long-term consequences for quality of life and functioning [14–16], aspects that are also impacted in those who are NEET. Yet, little is known about the profile of NEET individuals and how it differs from that of vocationally active individuals when they present at early intervention services and even prior to the onset of psychosis.

It has been suggested that NEET youths may not access mental health services or seek them late because of marginalisation and disengagement from systems [9, 17, 18]. Despite focusing on treatment delay for over 25 years [19–21], first-episode psychosis research has never examined these delays and care pathways among NEET youths. Such an examination is imperative if we are to know whether and how the early signs and symptoms preceding a first episode of psychosis (some of which constitute the prodrome) and the onset of psychosis go undetected among NEET youths, potentially worsening their illness and functioning trajectories.

Our aims were therefore to: (1) Document the prevalence of NEET status in a first-episode psychosis sample at a catchment-based early intervention service. (2) Investigate baseline differences between NEET and non-NEET groups in terms of symptomatology (positive and negative symptoms). (3) Examine whether NEET individuals had different pre-psychosis illness courses (i.e., premorbid adjustment and prodrome) and experienced longer treatment delays (duration of untreated psychosis or DUP) than their vocationally active counterparts. We also examined group differences in sources of delays, including delays in seeking help or the number of help-seeking contacts, and systemic delays in being referred to specialized care once help was sought.

## Methods

### Setting

This study was undertaken at a publicly funded specialized early intervention service, the Prevention and Early Intervention Program for Psychosis (PEPP), in Montreal, Canada. PEPP offers a 2-year program of services to persons experiencing first-episode psychosis [22]. Referrals are accepted from patients themselves or their families, schools, general practitioners, outpatient clinics, inpatient units or general or psychiatric emergency departments. Eligibility criteria are ages 14–35, diagnosis of affective or non-affective psychosis that is not substance-induced or caused by organic disorders, having received antipsychotic medications for no more than 30 days, and an IQ above 70. Patients with concurrent substance use disorders are included. Treatment comprises assertive case management, psychosocial interventions like cognitive-behavioural therapy and pharmacological management. PEPP is the only service of its kind in a catchment of about 300,000, making our sample close to a treated incidence sample. The present sample is derived from a study of first-episode psychosis course and outcomes, approved by the research ethics board of the Douglas Mental Health University Institute. All participants provided written informed consent, except assenting minors for whom consent was also sought from a parent/guardian. Data on NEET status were collected during baseline interviews.

### Sample

662 patients met PEPP’s inclusion criteria during the study’s 2003–2015 timeframe. Although PEPP serves individuals aged 14–35, only patients aged 15–29 were included in this study to be consistent with OECD [5] and Statistics Canada [23] conceptions of NEET. This reduced our potential participant pool to 550. Of those, 476 consented and data on NEET status were missing for 60 participants, leaving a final sample of 416.

### Baseline measures

NEET status was assessed using an item from the Strauss-Carpenter Scale [24]. It queried occupational/vocational functioning over the 12-month period preceding entry. Participants were classified as NEET or non-NEET. They were considered NEET if they were not employed (whether full- or part-time) and not in school at all, or if they were employed/in school for less than half the time in the past year. Those who did not meet these conditions were considered non-NEET. This definition of NEET is stricter than the OECD’s, whose timeframe is 1 reference week. We adopted this approach to include individuals who were vocationally disengaged for a substantial period, and not only immediately before entering treatment.

Demographic variables included age, gender, relationship status (coded either as single *or* as being in a relationship), living situation (coded either as living with family, spouse or friends, *or* as living alone, in group/nursing home or homeless), education (coded either as having completed high school *or* as having not completed high school), and visible minority status (coded either as non-white and non-Aboriginal, *or* as white) [25]. The Social Deprivation Index and Material Deprivation Index [26] of participants’ neighbourhoods (i.e., census-based geographic area) were used as proxy measures of socioeconomic status. Primary diagnosis (schizophrenia-spectrum or affective psychoses) and secondary diagnosis (presence or absence of substance abuse/dependence) were determined by the Structured Clinical Interview for DSM-IV (SCID-IV) [27], administered by trained staff within 3 months of entry. SCID-IV diagnoses were recorded based on consensus between the interviewer and the research team, including one experienced psychiatrist. Symptoms were measured using the Scale for the Assessment of Positive Symptoms (SAPS) [28], the Scale for the Assessment of Negative Symptoms (SANS) [29] and the Calgary Depression Scale for Schizophrenia (CDSS) [30]. Symptom assessments were done by trained staff who had achieved high inter-rater reliability rates (intra-class correlation range = 0.75–0.92). Total scores were calculated as the sum of all items for SAPS and CDSS, excluding the global items for SAPS. SANS total score was calculated as the sum of all items except the global ones, three attention-related items, and an item called “impersistence at work or school” to avoid overlap with NEET status.

The Circumstances of Onset and Relapse Schedule (CORS) [20], a semi-structured interview regarding lifetime history of illness, treatment delays and pathways to care before the onset of the presenting psychotic episode, was administered. CORS interviews were generally conducted with patients and their family members. Information was also collected from medical records and other sources to reconstruct timelines and estimate the following dates: date of first identifiable psychiatric change (non-psychotic), date of prodrome onset (change contiguous with the first psychotic episode), date of first psychotic episode, and date of commencement of first adequate treatment. The first psychiatric change was carefully distinguished from lifelong behaviour patterns (e.g., always having been withdrawn) and symptoms associated with longstanding childhood-onset conditions (e.g., attention deficit hyperactivity disorder). Adequate treatment was defined as taking antipsychotic medication for 1 month or until significant response, whichever came first. CORS data were used to determine key dates through consensus between the interviewer and the research team, including one experienced psychiatrist. Any discrepancy between various information sources was resolved by consensus.

The study included the following variables of interest from the CORS:

1. Duration of untreated psychosis (DUP), i.e., the time in weeks between the onset of the first psychotic episode and the start of adequate treatment. We separately estimated the help-seeking and referral components of DUP [31]. DUP help-seeking is the time from the onset of the psychotic episode until the first mental healthcare contact. DUP referral is the time from the first mental healthcare contact until referral to PEPP.
2. Duration of untreated illness (DUI), i.e., the time in weeks from the onset of the first psychiatric change to the start of adequate treatment.
3. Length of prodrome, i.e., the time in weeks between the onset of the prodrome and the onset of psychosis. The prodrome was defined as including non-psychotic symptoms, subthreshold psychotic symptoms and/or changes in behaviour, any or all of which were contiguous with the onset of psychosis.
4. Pathways to care, i.e., the number and type of mental health contacts from the first psychiatric change until entry into PEPP.

The 3-month training for administering the CORS included orientation, rating videotapes, role play, and conducting the CORS under supervision. To establish and maintain inter-rater reliability of the CORS, experienced raters (3–8) independently assessed randomly selected cases (3–20) on several occasions. High intra-class correlation coefficients (0.82–0.98) were always achieved in estimating delay indices and numbers of help-seeking contacts [32–34].

The Premorbid Adjustment Scale [35] was used to measure social and educational premorbid functioning during childhood (up to age 11), early adolescence (ages 12–15) and late adolescence (ages 16–18). Because the onset of psychotic disorders is usually in early adulthood, we did not include adjustment ratings for adulthood. “Premorbid” was defined as stopping 1 year before the first psychotic episode. Within each age range, information was collected from patients and/or family members on items regarding sociability, withdrawal and peer relationships, and scholastic performance and adaptation to school. We separately calculated scores for social and educational adjustment for each age range.

### Statistical analysis

Descriptive statistics are presented as proportions for count data and as means with standard deviations (SD) for continuous data. Independent samples *t*-tests (for continuous variables) and Pearson's chi-squared tests (for dichotomous variables) were used to assess group differences between NEET and non-NEET patients. All DUP variables were log-transformed because of high skewedness, and medians were also reported. Multivariable linear regression was applied to test the influence of NEET status on DUP, accounting for other known predictors: age at onset, gender, visible minority status, diagnosis, positive and negative symptoms, substance use diagnosis and levels of depression.

## Results

Non-participants (*n* = 60), i.e., those with missing NEET status data, differed from participants (*n* = 416) only on age [age at PEPP entry = 21.68 (SD 4.00), 22.71 (SD 3.61), respectively, *t*(474) = − 2.03, *p* = 0.043] and baseline SAPS score [SAPS = 38.02 (SD 14.70), 33.83 (SD 14.91), respectively, *t*(460) = 1.98, *p* = 0.049] and not on any other demographic or clinical characteristics.

### Aim 1: baseline prevalence of NEET status

39.18% of the sample (*n* = 163) were NEET upon entry into treatment.

### Aim 2: baseline illness characteristics of NEET vs. non-NEET patients

Demographic and clinical characteristics of non-NEET (*n* = 253, 60.82%) vs. NEET participants are presented in Table 1. NEET participants were likelier to be male and have a diagnosis of schizophrenia-spectrum rather than affective psychosis. Considering only those participants who were 18 or older (the age by which the majority of youths in Quebec complete high school), NEET youths were 31% less likely to have completed high school at the time of entry into treatment. The NEET group also had significantly higher levels of negative symptoms, even after having removed the SANS item for impersistence in work/school. NEET youths had 34% higher negative symptoms scores than non-NEET youths. The NEET group’s higher negative symptoms were also not attributable to them having been more depressed at admission.

**Table 1:** Demographic and clinical characteristics upon entry into treatment

Variable	Non-NEET at baseline	NEET at baseline	*X* ^2^	*p*
	*N* (%)	*N* (%)		
Gender (male)	171 (68%)	126 (77%)	4.58	**0.035**
Marital status (single)	231 (92%)	153 (94%)	0.88	0.432
Completed high school (age 18 or higher at PEPP entry^a^)	164 (78%)	80 (54%)	23.10	**0.000**
Visible minority	95 (39%)	56 (36%)	0.28	0.672
Living alone	35 (15%)	34 (21%)	3.23	0.080
Primary diagnosis (schizophrenia-spectrum)	171 (69%)	128 (79%)	4.55	**0.041**
Secondary diagnosis (substance abuse/dependence)	126 (53%)	95 (63%)	3.45	0.075

	Mean (SD)	Mean (SD)	*t*	*p*
Age at onset of FEP	21.80 (3.89)	21.94 (3.55)	− 0.35	0.723
Social Deprivation Index^b^	75.49 (20.45)	76.64 (18.40)	− 0.55	0.584
Material Deprivation Index^b^	60.70 (29.48)	63.54 (31.53)	− 0.88	0.377
SAPS total score	34.08 (14.80)	33.45 (15.11)	0.42	0.675
SANS total score	18.92 (12.42)	25.33 (12.94)	− 4.99	**0.000**
CDSS total score	5.22 (4.91)	4.27 (4.59)	1.94	0.054

Bold indicates significant findings

SD, standard deviation; FEP, first-episode psychosis; SAPS, Scale for the Assessment of Positive Symptoms, total score minus global items ranges from 0 to 150; SANS, Scale for the Assessment of Negative Symptoms, total score ranges from 0 to 85 after removal of global items, and items for attention and impersistence at work or school; CDSS, Calgary Depression Scale for Schizophrenia, total score ranges from 0 to 27

^a^This analysis was completed including only 360 individuals, who were at least 18 or older at the time of entry into our program, which is the age by which the majority of individuals have completed high school in Quebec, and for whom data regarding high school status was available

^b^Centile-based scores, with higher scores indicating greater deprivation

There was no statistically significant difference between the NEET and non-NEET groups in terms of their levels of depressive symptoms at baseline. Perhaps counterintuitively, there was a trend for the non-NEET group to report higher levels of depression. Future research should more carefully measure and examine the association between depression and NEET status in psychosis.

### Aim 3: Prodrome, premorbid adjustment and treatment delays of NEET vs. non-NEET patients

#### Prodrome and premorbid adjustment

As seen in Table 2, the NEET group had significantly longer prodromes than the non-NEET group (median of 51.86 vs. 24.29 weeks). Although most individuals entering PEPP experienced a prodrome (88% of the sample), the first psychiatric change was also the beginning of the prodrome for NEET individuals (62%) more often than non-NEET individuals (45%). In other words, once NEET individuals experienced a clear psychiatric symptom, they were less likely to recover and more likely to continuously have psychiatric symptoms/difficulties until their first psychotic episode. NEET and non-NEET youths experienced their first psychiatric change at similar ages [mean of 17.51 (SD = 4.93) vs. mean of 17.32 (SD = 4.86) years, respectively, *t*(395) = 0.39, *p* = 0.698].

**Table 2:** Premorbid adjustment and prodromal length of NEET vs. non-NEET individuals

Variable	Non-NEET at baseline	NEET at baseline	*t*	*p*
	Mean (SD)	Mean (SD)		
PAS childhood social score	0.18 (0.19)	0.22 (0.23)	− 1.45	0.150
PAS childhood education score	0.24 (0.19)	0.26 (0.18)	− 1.21	0.227
PAS early adolescence social score	0.23 (0.21)	0.25 (0.25)	− 0.68	0.500
PAS early adolescence education score	0.32 (0.25)	0.37 (0.22)	− 1.65	0.100
PAS late adolescence social score	0.23 (0.22)	0.30 (0.28)	− 2.13	**0.035**
PAS late adolescence education score	0.36 (0.26)	0.48 (0.26)	− 3.51	**0.001**
Length of prodrome in weeks^a^	1.33^c^ (0.79) Median = 24.29	1.55^c^ (0.77) Median = 51.86	− 2.81	**0.005**

	*N* (%)	*N* (%)	*X* ^2^	*p*
First psychiatric symptom is beginning of prodrome^b^	108 (45%)	96 (62%)	10.35	**0.001**

Bold indicates significant findings

PAS, Premorbid Adjustment Scale, total score ranges from 0 to 1 with lower scores indicating better adjustment

^a^Range = 0–931 for non-NEET group and 0–965 for NEET group

^b^ *n* for non-NEET group = 240; *n* for NEET group = 156

^c^Log-transformed values

Furthermore, compared to non-NEET individuals, NEET individuals had similar social and educational premorbid adjustment in childhood and early adolescence, but significantly lower social and educational premorbid adjustment in late adolescence (ages 16–18), which corresponds on average to the commencement of their prodrome. NEET youths’ social and educational premorbid adjustment scores in late adolescence were significantly lower by 30% and 33%, respectively, than those of their non-NEET counterparts. By excluding the year prior to the onset of psychosis from the assessment of premorbid adjustment, we eliminated the potential confound of threshold psychotic symptoms contributing to lowered adjustment. Moreover, given that the average age of onset in our sample was 21.86 (SD = 3.76) years, the likelihood that the onset of psychosis may have impacted adjustment in late adolescence is negligible.

#### Treatment delays

For the entire sample, median DUP was 15.57 weeks (range: 0–1011.57) and median DUI was 198.29 weeks (range 0.14–1283.86). As shown in Table 3, NEET individuals had a significantly longer DUP than non-NEET individuals, a median difference of 5.64 weeks. DUI did not differ significantly across the two groups. The regression analysis with log-transformed DUP onset as dependent variable yielded significant results; *F*(9,330) = 9.57, *p* = 0.000, adjusted *R*^2^ = 0.19 (see Table 4). Being NEET, along with being younger at psychosis onset, having a schizophrenia-spectrum diagnosis and having higher levels of depression, were independently associated with longer DUPs. On average, NEET participants’ DUP was 23% longer than that of the non-NEET group. Gender, visible minority status, severity of baseline positive and negative symptoms and having a substance use diagnosis at baseline were not associated with DUP.

**Table 3:** Treatment delays and course prior to entering PEPP

Variable	Non-NEET at baseline	NEET at baseline	*t*	*p*
	Mean (SD)	Mean (SD)		
DUP (weeks)	1.15^a^ (0.63) Median = 13.00 Range = 0–1011.57	1.35^a^ (0.65) Median = 18.64 Range = 0–455.57	− 2.94	**0.003**
DUI (weeks)	268.09 (248.18) Median = 193.57 Range = 1.00–1047.57	279.97 (256.07) Median = 218.29 Range = 0.14–1283.86	− 0.46	0.650
DUP help-seeking (weeks)	0.84^a^ (0.64) Median = 4.50 Range = 0–327.86	0.94^a^(0.68) Median = 7.14 Range = 0–286.14	− 1.57	0.118
DUP referral (weeks)	0.54^a^ (0.60) Median = 0.86 Range = − 13.57 to 313.43	0.67^a^ (0.68) Median = 1.71 Range = − 3 to 437.57	− 2.10	**0.036**
Total number of mental health contacts from first psychiatric change until PEPP entry	4.36 (2.21)	5.12 (2.86)	− 2.55	**0.012**

	*N* (%)	*N* (%)	*Χ* ^2^	*p*
Type of first contact			0.002	1.000
Physician	121 (65%)	86 (66%)		
Non-physician	64 (35%)	45 (34%)		
Source of referral to PEPP			1.48	0.477
Emergency	126 (51%)	86 (53%)		
Physician	88 (36%)	49 (30%)		
Non-physician	34 (14%)	27 (17%)		

Bold indicates significant findings

PEPP, Prevention and Early Intervention Program for Psychosis; DUP, duration of untreated psychosis; DUI, duration of untreated illness

^a^Log-transformed values

**Table 4:** Regression analysis predicting log-transformed duration of untreated psychosis (DUP)

Variable	*B*	SE	Standardized beta	*t*	*p*	95% confidence interval for *B*	
						Lower bound	Upper bound
NEET status	0.21	0.07	0.16	3.10	**0.002**	0.08	0.34
Age at onset of FEP	− 0.04	0.01	− 0.21	− 4.22	**0.000**	− 0.05	− 0.02
Gender	− 0.09	0.07	− 0.06	− 1.19	0.234	− 0.23	0.06
Visible minority status	− 0.03	0.07	− 0.03	− 0.49	0.627	− 0.16	0.10
Schizophrenia spectrum vs. affective Psychosis	− 0.50	0.07	− 0.35	− 6.83	**0.000**	− 0.64	− 0.35
SAPS at baseline	− 0.001	0.002	− 0.03	− 0.58	0.562	− 0.01	0.003
SANS at baseline	− 0.001	0.003	− 0.02	− 0.43	0.666	− 0.01	0.004
Substance use and dependence diagnosis at baseline	0.04	0.07	0.03	0.63	0.530	− 0.09	0.18
CDSS at baseline	0.01	0.01	0.11	2.05	**0.041**	0.001	0.03

Bold indicates significant findings

FEP, first-episode psychosis; SAPS, Scale for the Assessment of Positive Symptoms; SANS, Scale for the Assessment of Negative Symptoms; CDSS, Calgary Depression Scale for Schizophrenia

Additional analyses (see Table 3) were undertaken to investigate reasons for the longer DUP.

1. *Following the onset of psychosis, did the NEET group seek help after longer delays (i.e., have longer help-seeking delays) or were they less likely to be promptly referred to early intervention once they sought help (i.e., have longer referral delays) or both*? For the entire sample, median help-seeking delay was 5.71 weeks (range 0–327.86) and median referral delay was 1 week (range − 13.57 to 437.57; negative values represent patients referred from within PEPP’s sub-clinic for youths at ultra-high risk for psychosis). The NEET group had significantly longer referral delays than the non-NEET group (median of 1.71 and 0.86 weeks, respectively) but were not significantly different in terms of their help-seeking delays. Consistent with this, we found no evidence that NEET individuals sought help any less than their non-NEET counterparts. On the contrary, they made significantly more help-seeking attempts before entering PEPP (5.12 vs. 4.36 mean contacts for NEET vs. non-NEET, respectively).
2. *Do NEET individuals seek help from dissimilar sources than non-NEET individuals, contributing to their referral delay?* Based on our previous work [31], we hypothesized that more NEET individuals sought help from non-medical professionals (psychologists, school counsellors, clergy, addiction services, etc.) than from physicians, thus experiencing longer referral delays. However, we found that the first mental health contact for most NEET and non-NEET individuals was a physician. About half the sample was referred by emergency services, with no difference between the groups in who referred them to PEPP (emergency services, physician or non-physician). Finally, before entering PEPP, both groups sought help more frequently from medical rather than non-medical sources, with NEET individuals making significantly more medical (but not non-medical) help-seeking contacts than non-NEET individuals [NEET = 4.27 and non-NEET = 3.64 mean medical contacts; *t*(218) = 2.55, *p* = 0.012]. Further examination of the mean number of contacts by type of medical source revealed that the most-contacted sources were emergency services (NEET = 2.26, non-NEET = 1.83), followed by psychiatrists/psychiatric services (NEET = 2.03; non-NEET = 1.80), followed by primary care (NEET = 1.53, non-NEET = 1.21), and a few sources classified as “other” (e.g., neurologist) (NEET = 1.18, non-NEET = 1.00).

## Discussion

Nearly 4 in 10 persons entering our early intervention service had been NEET for 6 months or longer. This is thrice as high as the general NEET rate in Canada (13%) [23] and in  OECD countries (13.9%) [5]. This finding is consistent with reports of a heightened association between mental disorders and NEET status [4, 10, 11, 36, 37]. It builds on previous psychosis research that has noted school/work status to be impacted at entry into treatment and even in the prodromal course [12, 13, 38].

In our study, non-NEET individuals usually developed psychosis after several periods (or at least one) of wellness following their first notable psychiatric change. NEET youths, on the other hand, were 38% more likely to remain unwell after their first psychiatric change at around age 17. Their first psychiatric change more often marked the beginning of their prodrome, which was longer than that of non-NEET youths. In late adolescence, the NEET group began exhibiting lower social and educational premorbid adjustment than the non-NEET group, despite having had similar adjustment levels in childhood and early adolescence. Thus, from late adolescence, the group that entered treatment as NEET began following a distinct trajectory of persistent mental health problems and functional decline that built up towards an eventual intersection of psychosis and NEET status. The functional decline of NEET youths was evinced by their NEET status at entry, their lower rates of high school completion, and their lower premorbid adjustment, particularly in the educational domain.

Because the association between functional impairments and mental ill-health is bi-directional [9, 10, 13] and because our study was retrospective, we can draw several conclusions—that pre-psychosis psychiatric symptoms exacerbate functional decline, that functional impairments increase the risk for psychiatric symptoms, or that the two effects operate contiguously, simultaneously or iteratively. Regardless of the directionality of the effect, individuals who enter early intervention services as NEET may be a discrete sub-group in terms of both their pre-onset course and their baseline characteristics.

### Baseline illness characteristics of NEET individuals with psychosis

NEET individuals presented with substantially higher levels of negative symptoms than non-NEET individuals. One explanation is that being NEET increases risk for negative symptoms. NEET youths had a longer DUP, which has previously been associated with more severe baseline negative symptoms [39]. Furthermore, the phenomenological and behavioural features that are known to be associated with being NEET (social withdrawal, poor motivation, etc.) bear the same signature as the negative symptoms of psychosis [37, 40, 41]. It may therefore also be the case that NEET individuals were already experiencing negative symptoms before entering early intervention services [42].

Furthermore, our study suggests that both pre-onset NEET status and negative symptom-like phenomenology may be part of a long, insidious latent process marked by persistent mental health symptoms and functional decline. Another manifestation of this distinctness of the NEET group is that they were likelier to present with schizophrenia-spectrum, rather than affective, psychoses. Our NEET sub-group bears some resemblance to the sub-sample of Häfner’s Age, Beginning, Course (ABC) study with unspecific and/or negative prodromal symptoms [12]. Häfner reported that the number of non-fulfilled social roles at onset (e.g., work, school), along with socially adverse behaviour (e.g., substance use) at first admission were the only significant predictors of this sub-group’s ability to earn a living 5 years later. Traditional predictors, like age of onset, gender, symptomatology and acute vs. insidious onset, only acted through these two variables [12]. Although not statistically significant, there was a trend for the NEET group in our study to have a higher likelihood of a substance use diagnosis at baseline. In addition to being in line with Hafner’s results, our finding in this regard is also consistent with other research pointing to the association between substance misuse and NEET status [4, 36, 43].

### Treatment delays among NEET individuals with psychosis

Given that PEPP patients' median DUP of 15.57 weeks is well below DUPs reported in international data [21, 44–46], it is even more noteworthy that the DUP was prolonged for NEET individuals. Strikingly, the NEET group’s longer treatment delay was attributable not to delayed or fewer help-seeking attempts, but to delays in being referred to and accessing early intervention after entering the health and social service system. The type (medical or non-medical) of the first mental healthcare contact, types of help-seeking contacts, and sources of referral to the early intervention service did not explain the NEET group’s longer DUP. Consistent with prior findings [31], over half the patients in our sample—NEET and non-NEET—were referred from the emergency department, suggesting that emergency services, despite their general undesirability, represent an expedient access portal in a system fraught with waiting lists and too few family doctors.

Before entering PEPP, NEET individuals made more help-seeking contacts than the non-NEET group, particularly with physicians. Yet, many of them were left untreated even when actively psychotic. Over 65% of them were not adequately treated for their psychosis for more than 3 months. Comparatively, the median for the non-NEET group was 13.00 weeks, i.e., close to 3 months. The 3-month cut-off is significant because it corresponds to DUP targets recommended by an influential international consensus statement [47].

Psychosis among NEET individuals seems to have been under-recognized or recognized late even though they presented with equal levels of positive symptoms as non-NEET individuals. NEET individuals present with more severe negative symptoms, a longer prodrome, and poorer pre-onset functioning starting in late adolescence. Against this insidious and burdensome backdrop, psychotic symptoms may not be detected by service providers or may not be attributed to psychosis. On the other hand, in non-NEET individuals, who have shorter prodromes with less pre-psychosis functional decline, psychotic symptoms may stand out more and trigger timelier referrals to early intervention services. This suggests that referral sources—including physicians—have trouble discerning psychosis when it is overlaid on a long history of symptoms and difficulties for which NEET individuals may have already sought help. Finally, one cannot discount the possibility that NEET individuals’ poor disclosure or inconsistent engagement with service providers and service providers’ actual or perceived biases against socioeconomically marginalized individuals [48, 49] may contribute to their longer DUPs.

### Demographic characteristics of NEET individuals with psychosis

In our sample, youths who were NEET were more likely to be males than females. This is significant because although psychosis afflicts more men than women, NEET status itself is equally distributed among men and women in the general Canadian youth population [23]. In Canada, 23% of NEET youths had not completed high school by age 18. In our sample, 46% of NEET individuals had not finished high school by age 18, indicating that psychosis or its pre-onset course may exacerbate the functional difficulties that NEET youths are known to face. Our results also suggest the role of low educational attainment in contributing to NEET status and highlight the need for early educational supports with a view to reducing functional decline trajectories among youths [50].

Much evidence highlights lower socioeconomic status as a risk factor for becoming NEET [51, 52]. However, we found no difference between the NEET and non-NEET groups’ social and material deprivation. We used neighbourhood-level and not individual-level indices of deprivation. Being catchment-based, our service caters to patients from similar neighbourhoods, most of whom (NEET and non-NEET) come from the lowest two quartiles of social and material deprivation (90.6 and 68.6%, respectively). The low variance in these deprivation indices may have limited our ability to detect socioeconomic differences between the NEET and non-NEET groups.

### Limitations

We had no information on exactly how long participants had been NEET. The NEET group could have included individuals who had been NEET for longer than the pre-entry year we considered. In the ABC sample, for instance, Maurer and Häfner noted “loss of interest in workplace” about 24.4 months before first admission [12]. Had our participants been NEET for longer, they may have had more profound consequences (e.g., more severe negative symptoms). Conversely, being NEET for a shorter duration may have corresponded with a high-risk state or with the onset of psychosis. The latter is less likely as our median DUP was 15.57 weeks and we defined NEET to include a much longer timeframe.

We did not record why youths were NEET. Adverse social contexts (e.g., having been in child welfare or having been inadequately prepared for transitioning out of it) may have increased their vulnerability to both becoming NEET and to having their psychosis detected late. Also, the NEET group may have included those who were looking for work/school opportunities, not looking for work/school, voluntarily NEET, and taking a break. This inclusive conception of NEET is consistent with prior literature [4, 23].

We included data from over 87% of patients entering our service who were aged 15–29. We noted that those excluded had higher levels of positive symptoms and were younger. Those excluded may also have been NEET and/or experienced longer DUPs. If so, our findings underestimate the impact of being NEET on DUP. Conversely, those excluded may have experienced an extremely short pathway despite being NEET. Irrespective of the potential for bias introduced by the exclusion of this sub-group, the association we found between being NEET and having a longer DUP is noteworthy.

### Strengths

This paper is one of less than a handful of data-driven investigations of the intersection between NEET status and psychosis. Its strengths include the representativeness of its catchment-based sample; a well-characterized sample with rigorous measures of diagnosis, symptoms, premorbid adjustment and treatment delays; and careful estimation of pre-treatment employment/education status. To evaluate NEET status, our study used a longer timeframe than the OECD (past week) to better capture youths facing more persistent vocational problems. We also did not classify those who were rearing children as NEET. We believe that this a strength over most published studies [4, 11, 23] which classify youths as NEET if they are not in education, employment or training, independent of their parental obligations.

DUP, DUI, prodromal length, pathways to care and premorbid adjustment were assessed by well-trained raters with high inter-rater reliability and were confirmed by consensus.

Our results suggest that a sizeable number of individuals with first-episode psychosis who enter early intervention services may be NEET. This is disconcerting given the known adverse longer-term consequences of youth disengagement from employment and education. In psychosis, baseline unemployment is known to translate into poor functioning even years later [12, 53–55].

Our systematic analytical approach revealed a distinct trajectory among NEET youths up to the point of entering early psychosis services—poorer adjustment and mental health problems beginning in late adolescence, accompanied by multiple help-seeking contacts and a longer DUP. This analytical approach can be adopted and extended in various international settings to further investigate and mitigate the intersection between NEET status and mental health problems, including psychosis. Our findings make the case for early case identification interventions targeted at NEET youths to reduce their treatment delays and arrest potentially downward spirals of illness and functional decline. They also highlight the need for earlier, broader-spectrum interventions that address youths’ educational and occupational concerns in addition to their mental health issues.
